# Surgical Staging of Locally Advanced Cervical Cancer: Current Status and Research Progress

**DOI:** 10.3389/fonc.2022.940807

**Published:** 2022-07-06

**Authors:** He Zhang, Weimin Kong, Shuning Chen, Xiaoling Zhao, Dan Luo, Yunkai Xie

**Affiliations:** Department of Gynecological Oncology, Beijing Obstetrics and Gynecology Hospital, Capital Medical University, Beijing Maternal and Child Health Care Hospital, Beijing, China

**Keywords:** surgical staging, locally advanced cervical cancer, concurrent radiotherapy, research progress, prognosis

## Abstract

Locally advanced cervical cancer (LACC) has large localized lesions, high recurrence and metastasis rate under standard treatment, and low survival rate. The current guidelines still use concurrent radiotherapy as the gold standard of treatment for locally advanced cervical cancer. Several recent studies have shown that surgical staging has higher accuracy in determining metastasis in the para-aortic lymph nodes, bringing survival benefits to some patients. However, the indications for surgical staging and whether surgical staging can improve prognosis are still controversial. We will review the current status and research progress of surgical staging for locally advanced cervical cancer.

Cervical cancer is one of the most common malignancies of the female reproductive system and poses a severe threat to women’s health. It is estimated that there will be about 14,100 new cases of cervical cancer in 2022 across the United States (1). With the prevalence of cervical cancer screening, most patients are diagnosed at an early stage of the disease and have a better prognosis. However, there are still a significant number of patients who are diagnosed at an advanced stage due to a lack of awareness of cervical cancer screening. According to the definition of locally advanced cervical cancer by the National Comprehensive Cancer Network (NCCN) guidelines, locally advanced cervical cancer is cervical cancer with FIGO 2018 stage IIB to IVA (2). Patients with locally advanced cervical cancer have a high incidence of lymph node metastasis. Lymph node metastasis directly affects the patient’s staging, the development of radiotherapy scope, and prognosis. Imaging is the conventional method to determine whether lymph node metastasis exists in locally advanced cervical cancer patients. Still, there are limitations, especially in determining whether lymph nodes adjacent to the abdominal aorta are metastatic. In recent years, surgical staging of locally advanced cervical cancer has been applied in clinical practice, a surgical method to determine the presence of lymph node metastasis by resecting the common iliac and low para-aortic lymph nodes (PALN) before concurrent radiotherapy. Surgical staging for these lymph nodes allows for more accurate determination of lymph node metastases and thus the extent of the external irradiation field. However, there are still controversies about the indications of surgical staging and whether it can improve the prognosis. This review will discuss the history of surgical staging, the prognostic impact of surgical staging, and the implementation of surgical staging.

## Overview of LACC: Diagnosis and Treatment

Patients with locally advanced cervical cancer have high rates of lymph node metastasis, parametrial involvement, the incidence of vascular infiltration, and other moderate to high-risk recurrence factors. Their 5-year overall survival (OS) rates are only 50% to 60% (3). According to the 2016 American Cancer Society (ACS), the 5-year overall survival rate for stage IIB cervical cancer is about 58%, stage IIIA is 35%, stage IIIB is 30%, and stage IVA is about 16% (4). The 2022 NCCN guidelines recommend a treatment regimen for locally advanced cervical cancer that starts with imaging. Patients with negative imaging are treated with pelvic field radiotherapy + cisplatin-containing concurrent chemotherapy + vaginal afterloading radiotherapy (5). Patients with positive lymph nodes on imaging are classified as stage IIIC according to the FIGO2018 cervical cancer staging. Only pelvic lymph node metastasis is considered stage IIIC1, and the presence of para-abdominal aortic lymph node metastasis is considered stage IIIC2.

The change in the staging of lymph node metastases in FIGO2018 indicates that pelvic and para-aortic lymph node metastases significantly impact patient prognosis. The staging should also indicate the method of diagnosis of lymph node metastasis, with remarks (r) for diagnosis by imaging and remarks (p) for diagnosis by surgical staging (6). The NCCN guidelines also recommend that patients with metastases to the parietal common iliac artery and para-aortic lymph nodes should be treated with extended field radiotherapy. Some investigators have suggested prophylactic extension field radiotherapy for patients with metastases in the para-aortic lymph nodes (7). Still, there is no evidence that prophylactic extension field radiotherapy improves survival in all locally advanced cervical cancer patients. This approach increases the likelihood of side effects and complications. Therefore, assessment of metastases in the para-aortic lymph nodes is of great importance for more precise treatment planning. In addition to conventional imaging, direct surgical staging can also be performed to assess the lymph node status and complement extended field radiotherapy or systemic therapy depending on intraoperative lymph nodes and distant lymph node metastases.

### Lymphatic Metastases in LACC: Should We Trust Imaging?

The pelvic and para-aortic lymph node metastasis rates in patients with locally advanced cervical cancer have been reported to be approximately 50% and 30%, respectively (8). Among them, the prognosis is most significantly affected by the metastasis to the para-aortic lymph nodes. And pelvic lymph node metastasis is the most important factor associated with para-aortic lymph node metastasis in the abdomen (8). It has been reported in the literature that 10%-25% of patients with cervical cancer have metastasis to the para-aortic lymph nodes. The more advanced the clinical stage is, the higher the rate of metastasis to the para-aortic lymph nodes, with 16%, 29%, and 36% occurring in stages IIB, III, and IVA, respectively (9). Imaging is a routine method to determine the occurrence of lymph node metastasis in patients with locally advanced cervical cancer. Traditional methods, such as imaging techniques like magnetic resonance imaging (MRI) or computed tomography (CT), suffer from a high false-negative rate. Positron emission tomography/computed tomography (PET/CT) is recommended in the guidelines for the evaluation of lymph nodes and distant metastases in locally advanced cervical cancer. A review published by Smits et al. in 2014 that included 22 studies suggested a false-negative rate of 9-35% for PALN, 4-11% for PET, and 6-15% for PET/CT in patients with locally advanced cervical cancer who underwent preoperative CT scan and/or MRI (9). A recently published Meta-analysis from 39 studies showed that PET/CT. The sensitivity of PET/CT, CT, and MRI in the diagnosis of lymph node metastasis was 82%, 50%, and 56%, respectively, and the specificity was 95%, 90%, and 91%, respectively (10). This study suggests that there is an urgent need to improve the sensitivity of diagnosing lymph node metastasis, and the search for more sensitive methods for the diagnosis of lymph node metastasis deserves further research and investigation.

### Radiotherapy in LACC: Current Status and Current Dilemmas

The conventional extracorporeal irradiation for cervical cancer generally does not include the para-aortic area. However, a high rate of para-aortic lymph node metastasis in locally advanced cervical cancer is common after treatment recurrence. Clearly, for patients with locally advanced cervical cancer with metastasis in the para-aortic lymph nodes, radiotherapy confined to the pelvic cavity alone is not sufficient. Recent studies have shown that expanding the scope of radiotherapy can improve the overall survival of patients with locally advanced cervical cancer (11). The 2022 NCCN guidelines (5) recommend extended-field radiotherapy (EFRT) for locally advanced patients with metastases in the para-aortic lymph nodes or high-grade common iliac lymph nodes to control or eliminate metastatic lesions in the para-aortic lymph node metastases, reducing regional recurrence and distant metastases, improving tumor local control rate and prolong overall survival time. The guidelines emphasize the defined scope of extended field radiotherapy, and prophylactic extended field radiotherapy can be considered to extend to the level of the presacral lymph nodes to the common iliac artery when the pelvic lymph nodes are involved; when patients develop metastatic involvement of the common iliac lymph nodes, intensity-modulated radiotherapy can be considered to extend to the level of the para-aortic lymph nodes to the renal vessels (5).

Although extended field radiotherapy for locally advanced cervical cancer has been recommended in the guidelines, the complications of prophylactic extended field radiotherapy are much higher than pelvic radiotherapy. According to the results of the RTOG 79-20 study, the 10-year incidence of grade 4-5 toxic events due to prophylactic EFRT and pelvic radiotherapy was 8% and 4%, respectively (11). Weighing the pros and cons, it is more important to identify patients with extra-pelvic lymph node metastases accurately. Imaging is non-invasive and simple, but diagnostic accuracy is poor and subject to error. Surgical staging enters the picture here. Surgical staging is more accurate for patients with lymph node metastases based on imaging staging and has the potential to help clinicians better assess the condition.

## Surgical Staging in Locally Advanced Cervical Cancer

### History of Surgical Staging

Currently, surgical staging of locally advanced cervical cancer has been performed for more than 20 years (12). Long-term clinical observations have revealed that 10-15% of patients with a negative diagnosis of para-aortic lymph nodes by conventional imaging methods such as PET/CT still have lymph node metastases found in pathological staging. Therefore, histopathological examination has long been the gold standard for identifying metastases in the para-aortic lymph nodes. Several studies published in the 1990s did not definitively confirm that surgical staging of locally advanced cervical cancer improves patient prognosis, and surgical staging is associated with a high rate of postoperative complications (12). New studies published in the last decade have again explored the efficacy of surgical staging in locally advanced cervical cancer. The benefit of surgical staging to patients currently depends mainly on the following aspects.

1. Whether intraoperative detection of metastasis in the para-aortic lymph nodes of the abdomen changes the subsequent treatment strategy and thus improves survival.
2. The impact of intraoperative lymph node dissection on patient prognosis.
3. The incidence of complications associated with surgical staging.
4. Whether there is a delay in the administration of concurrent radiotherapy after surgery compared to patients treated directly with concurrent radiotherapy.

### Impact of Surgical Staging on Prognosis

Studies on the prognostic impact of para-aortic lymph node dissection on the prognosis are numerous, and the conclusions are not entirely uniform. A systematic review of surgical staging published by Cochrane in 2013 did not find a survival benefit due to the inclusion of only one small trial with moderate bias. This study suggested that the assessment of whether to perform lymph node dissection in patients with locally advanced cervical cancer should be individualized (13). However, several recently published studies have favored surgical staging for locally advanced cervical cancer patients. Smits et al. conducted a systematic review of 22 studies on surgical staging of locally advanced cervical cancer (9). In 7-58% of these cases (mean 20%), treatment was improved by surgical staging, and patients’ disease-free survival and overall survival may have improved. The prospective randomized controlled trial UTERUS 11, presented at the 2019 International Gynecologic Oncology Society Annual Meeting (IGCS), included 240 patients with locally advanced cervical cancer who were randomized to receive either imaging staging or surgical staging (14). Although the study failed to show a significant difference in overall survival (OS) between surgical and imaging staging (95% CI: 0.48-1.05, p=0.084), surgical staging resulted in a higher cancer-specific survival rate in comparison (HR 0.61, 95% CI: 0.40-0.93, p=0.020). Also, surgical staging, especially the application of laparoscopic surgery, did not lead to significant delays in radiotherapy and a lower incidence of perioperative-related complications. Unfortunately, PET-CT was not routinely used as a diagnostic imaging method for patients in the UTERUS 11 study. Another randomized trial comparing PET-CT with surgical staging (PALDISC trial) is underway.

A retrospective study by Gold et al. analyzed 685 patients from three studies of the Gynecologic Oncology Group (GOG), of whom 550 patients had negative abdominal para-aortic lymph nodes confirmed by surgical staging and 130 patients had negative abdominal para-aortic lymph nodes confirmed by imaging. Although patients in the imaging staging group had milder disease and smaller tumors, patients in the surgical staging group had significantly higher disease-free survival (DFS) and overall survival (OS) than those in the imaging staging group. This difference was more significant in advanced disease (stages III-IV) (36.2% in the 4-year DFS surgical staging group vs. 48.9% in the radiation group and 40% in the 4-year OS surgical staging group vs. 54.3% in the radiation group). Also, patients in the pretreatment by imaging staging group had a higher rate of metastasis in the para-aortic lymph nodes after concurrent radiotherapy than patients in the surgical staging group (15).

### The Specific Implementation of Surgical Staging

#### Indication of Surgical Staging

There is controversy regarding the indications for surgical staging before concurrent radiotherapy for locally advanced cervical cancer. The 2020 British Gynaecological Cancer Society (BGCS) guidelines for cervical cancer recommend that patients with negative para-aortic lymph nodes on imaging may be considered for surgical staging before treatment (16). The 2017 European Society of Medical Oncology (ESMO) guidelines for cervical cancer recommend that surgical staging is feasible for patients with locally advanced cervical cancer. That staging is followed by a decision on the next treatment option based on lymph node metastasis (17). According to the 2022 NCCN guideline (18), the metastasis of the lymph nodes adjacent to the abdominal aorta should be thoroughly evaluated before surgical staging. PET/CT is the imaging gold standard for determining metastasis in the para-aortic lymph nodes. Patients with PET/CT indicating positive para-aortic lymph nodes (stage IIIC2r) need to receive EFRT + concurrent chemotherapy + endoluminal radiotherapy. We should focus on their pelvic lymph node metastasis in patients with PET/CT suggestive of negative para-aortic lymph nodes. Patients with negative pelvic lymph nodes have a lower risk of metastasis to the para-aortic lymph nodes, a lower potential overall survival benefit from surgical staging, and surgical staging is not recommended. Patients with PET/CT suggestive of pelvic lymph node metastasis (stage IIIC1r) have the potential for false-negative PET/CT para-aortic lymph nodes. They may be considered for surgical staging to assess for the presence of para-aortic and distant metastases (18). Surgical staging can also be used to remove enlarged lymph nodes that are difficult to clear with standard doses of radiotherapy (14). PET-CT is recommended for imaging to evaluate lymph node metastases. For areas where PET-CT is not available, CT may be considered. However, CT has a higher rate of false negatives and is more likely to result in missed metastases in the para-aortic lymph nodes.

#### Surgical Staging: Methods, Approaches, and Scope of Surgery

The route of surgical staging can be performed either transperitoneally or extraperitoneally (12). A systematic review of 19 studies showed that the rate of transperitoneal complications was higher for extraperitoneal surgical staging, but there were no significant differences between the two procedures in terms of intraoperative and postoperative complication rates (19).

Surgical modalities include open surgery, laparoscopic or robot-assisted laparoscopic surgery (20). Laparoscopic surgery is less invasive, has a faster recovery, and has less impact on subsequent treatments such as simultaneous radiotherapy, making it currently the treatment of choice. In recent years, newly developed laparoscopic-assisted robotic surgery can reduce bleeding, shorten surgery and hospitalization time, and decrease perioperative and postoperative complication rates. A single-center retrospective study by Loverix et al. compared patients with locally advanced cervical cancer who underwent surgical staging using laparoscopic and robotic assistance (21). The results showed no significant differences in 2-year progression-free survival (PFS) (P=0.472) and overall survival (P=0.749) between the two approaches, suggesting that robotic-assisted surgical staging leads to better perioperative outcomes and similar survival outcomes compared with laparoscopic surgical staging.

The extent of lymph node dissection in surgical staging is not universally agreed upon but varies from one literature to another. Clinicians may choose the extent of surgery based on imaging and intraoperative conditions. The NCCN guidelines suggest that the size of lymph node dissection should usually reach the level of the inferior mesenteric artery (IMA) (18), including mainly the common iliac lymph nodes and the para-aortic lymph nodes (up to the level of the inferior mesenteric artery). Suppose there is a high intraoperative suspicion of pelvic or para-aortic lymph node metastasis. In that case, the procedure can be extended to the level of the renal vessels of the abdominal aorta (4).

Finally, although para-aortic lymph node dissection in surgical staging is a well-defined and standardized procedure, anatomical abnormalities may lead to dangerous intraoperative complications (22, 23). Therefore, accurate preoperative imaging studies of the anatomy of structures such as blood vessels can help prevent unintended intraoperative injuries.

### Complications of Surgical Staging and Associated Radiotherapy Delays

Surgical staging currently suffers from complications and delays in simultaneous postoperative radiotherapy. It has been reported in the literature that the perioperative complication rate for surgical staging of locally advanced cervical cancer is approximately 5-24% (24), and concurrent pelvic and para-aortic lymph node dissection increases the incidence of complications (25). According to the UTERUS 11 study, the incidence of intraoperative complications (e.g., ureteral injury, inferior mesenteric artery bleeding, etc.) in locally advanced cervical cancer staged surgery was 1.6%, and the incidence of early postoperative complications (e.g., lymphatic cysts, intestinal adhesions, intestinal obstruction, etc.) was 7.6%, with no patients dying perioperatively (26). Lymphatic cysts and lymphedema are the most important complications after lymph node dissection, accounting for more than half of the postoperative complications of surgical staging (27, 28). Their treatment is mainly based on conservative treatment. Postoperative radiation therapy also increases the incidence of complications such as nausea, vomiting, and diarrhea (21). With the widespread implementation of laparoscopic surgery and robot-assisted laparoscopic surgery in recent years, related complications have significantly been reduced. Patients can start radiotherapy within 10-21 days after surgical staging. The proportion of patients with radiotherapy delayed for more than 30 days due to surgical complications was less than 5%, and no prognosis was found to be affected by radiotherapy delay due to surgical staging, nor was there a significant difference in radiotherapy delay between surgical and imaging staging (14).

### Treatment After Surgical Staging

Patients whose surgical staging suggests no positive para-aortic lymph nodes are given pelvic field radiotherapy + platinum-based concurrent chemotherapy + brachytherapy postoperatively according to the 2022 NCCN guidelines. Should patients with surgical staging suggestive of positive para-aortic lymph nodes be routinely treated with extended field radiotherapy and the above treatment? Existing studies have found that the size of positive para-aortic lymph nodes determines whether or not extended field radiotherapy is administered after surgical staging. Lymph nodes larger than 1.5 cm are considered macroscopic metastases and should be removed during surgical staging. Postoperative extended field radiotherapy in this subset of patients (stage IIIC2p) is not controversial (26). For micrometastatic disease less than 5 mm or found microscopically, it is controversial whether postoperative extended field radiotherapy should be administered (12). Leblanc et al. showed that patients with micrometastases and resected para-aortic lymph nodes who underwent postoperative extended field radiotherapy had the same survival rate as patients with negative lymph nodes who received pelvic irradiation alone (29). Gouy et al. reported three-year disease-free survival (DFS) rates of 74%, 69%, and 17% after extended-field radiotherapy in patients without abdominal para-aortic lymph node involvement and with abdominal para-aortic lymph node involvement less than and greater than 5 mm, respectively. This may be because the biological effect of simultaneous radiotherapy alone is sufficient to treat micrometastases smaller than 5 mm (30). The above study suggests that extended field radiotherapy should be routinely administered for macroscopic metastases; for micrometastases, no supplemental extended field radiotherapy can be considered after adequate evaluation (31).

## Conclusion

In summary, surgical staging for locally advanced cervical cancer is highly accurate for determining lymph node metastasis, can improve cancer-specific survival rates, and has low associated complications. After the metastasis of para-aortic lymph nodes is thoroughly evaluated by PET/CT, patients with positive pelvic lymph nodes but negative para-aortic lymph nodes can be considered for surgical staging to assess the presence of para-aortic lymph node metastasis. Surgical staging clarifies the scope of postoperative radiotherapy and reduces complications associated with extended field radiotherapy. The scope of surgical staging for lymph node dissection includes mainly the para-aortic lymph nodes (at the level of the inferior mesenteric artery) and the common iliac lymph nodes. Laparoscopic surgical staging is preferred, and robotic-assisted laparoscopic surgical staging can also be performed in areas where it is available. Surgical staging is worth promoting in the clinic. However, there are still many problems, such as the determination of surgical indications and the scope of surgery and the management of surgical complications, which need further research. Clinicians should select individualized treatment methods according to the patients’ own conditions of locally advanced cervical cancer so as to achieve precision medicine and achieve the purpose of improving clinical outcomes and the quality of life of patients with locally advanced cervical cancer.

## Author Contributions

HZ and WMK contributed to the conception of the study and wrote the manuscript. All authors contributed to the article and approved the submitted version.

## Conflict of Interest

The authors declare that the research was conducted in the absence of any commercial or financial relationships that could be construed as a potential conflict of interest.

## Publisher’s Note

All claims expressed in this article are solely those of the authors and do not necessarily represent those of their affiliated organizations, or those of the publisher, the editors and the reviewers. Any product that may be evaluated in this article, or claim that may be made by its manufacturer, is not guaranteed or endorsed by the publisher.
